# The diverging role of increasing wildfire smoke to ambient PM_2.5_ exposure disparity in California, 2006 to 2018

**DOI:** 10.1371/journal.pclm.0000796

**Published:** 2026-02-04

**Authors:** Jenny T. Nguyen, Joan A. Casey, Tarik Benmarhnia, Chen Chen

**Affiliations:** 1 Scripps Institution of Oceanography, University of California San Diego, La Jolla, California, United States of America; 2 Department of Environmental Health Sciences, Fielding School of Public Health, University of California Los Angeles, Los Angeles, California, United States of America; 3 Department of Environmental and Occupational Health Sciences, University of Washington School of Public Health, Seattle, Washington, United States of America; 4 Department of Epidemiology, University of Washington School of Public Health, Seattle, Washington, United States of America; 5 Irset Institut de Recherche en Santé, Environnement et Travail, UMR-S, Inserm, University of Rennes, EHESP, Rennes, France; 6 Department of Population Health Sciences, University of Utah, Salt Lake City, Utah, United States of America; 7 Huntsman Cancer Institute, University of Utah, Salt Lake City, Utah, United States of America

## Abstract

Exposure to ambient fine particulate matter (PM_2.5_) varies by structural determinants of health, through mechanisms such as racism and material deprivation. These disparities are well documented in the US across individual and community-level race and ethnicity (RE) and socioeconomic status (SES). Since 2000, California air quality has generally improved, and disparities have narrowed, tentatively attributed by previous studies to air regulations. In parallel, wildfires became major contributors to ambient PM_2.5_, with different exposure patterns from traditional emission sources. To explore wildfires’ contribution to exposure disparities, we tracked the temporal trend in total ambient PM_2.5_ exposure disparities in California from 2008 to 2006 and disentangled the role of wildfire smoke. We evaluated the population-weighted and rank-ordered temporal change in total, wildfire, and non-wildfire PM_2.5_ exposure across California census tracts and by RE and SES groups. We confirmed an absolute decrease in total PM_2.5_ over time and fluctuations in wildfire PM_2.5_ with peaks in 2008 and 2018. Census tracts with historically high total PM_2.5_ exposure in 2006 were less exposed in 2018, but this rank-ordered temporal change was mostly driven by increased wildfire PM_2.5_ in some tracts. Across the study period, community disparities in total PM_2.5_ existed among RE and SES groups, with higher exposure among socioeconomically disadvantaged and non-Hispanic Black and Hispanic populations. Community disparities in total PM_2.5_ narrowed from 2006 to 2018, yet these reductions were 9.4% to 59.5% attributable to increased wildfire PM_2.5_ exposure among socioeconomically affluent or non-Hispanic populations. In summary, wildfire PM_2.5_ has exaggerated the progress in reducing inequities in traditional sources of PM_2.5_, especially across racial groups and in years with severe wildfire like 2018. Additional targeted efforts are needed to reduce persistent inequities in PM_2.5_ exposure from traditional sources, which can be masked by increases in wildfire PM_2.5_ in an era of climate change.

## Introduction

Health burdens, which can impede people’s daily lives and wellbeing, are often distributed unevenly across space, ultimately leading to global and national health disparities [1] that span the socioeconomic gradient. Fine particulate matter (PM_2.5_), a major component of ambient air pollution, is a potential modifiable contributor to these health disparities. Total mass PM_2.5_ is an airborne and inhalable mixture of particles less than or equal to 2.5 μm in aerodynamic diameter. In 2021, ambient PM_2.5_ was ranked as the fourth highest mortality risk factor and contributed to 4.7 million premature deaths globally [2]. PM_2.5_ contributes to health disparities through differential exposure and differential effect (i.e., differential susceptibility) among groups, which are not mutually exclusive [3,4]. Locations of emission sources vary notably due to historical discriminatory housing and land use practices, regional climate, and the geophysical environment [5]. In the United States (US), communities of color and lower socioeconomic status (SES) experience higher exposures and bear larger health burdens from such exposures, compared to white or wealthy populations [6–8], which can be attributed to a combination of sociopolitical factors such as structural discrimination [9] and historical systemic racist policies [10]. As such, evaluating potential disparities in exposure to PM_2.5_ across sociodemographic factors could unveil potential drivers for health disparities and support targeted efforts to reduce PM_2.5_ exposure and related health burdens among disadvantaged subpopulations [5,9].

The Clean Air Act (CAA) was promulgated in the US in 1970 and led to major reductions in ambient PM_2.5_ concentrations nationally following its enactment [11]. However, reductions did not accrue equally across groups, which prompted the passage of regulatory actions such as Executive Order 12898, as signed by President William Clinton in 1994, to address inequities in exposure to environmental hazards [12]. Disparities in ambient PM_2.5_ exposure between white and Black populations declined from 2000 to 2015, which was attributed to the CAA’s larger impact on the historically most polluted areas, where more Black populations reside [13]. Despite decreases in total ambient PM_2.5_ concentrations and exposure disparities over time, the rank order of exposed areas has remained static, with the historically most exposed remaining the most exposed [5]. Moreover, compared to white and more affluent populations, certain vulnerable subpopulations, such as Hispanic and American Indian and less affluent populations, benefited less from relative emission reductions among traditionally dominant sources of PM_2.5_ (e.g., from industry and energy sectors) following the enactment of the CAA from 1970 to 2010 [14]. Overall, higher ambient PM_2.5_ exposure persisted among racial and ethnic (RE) minoritized groups and lower SES communities through 2016, despite absolute exposure reductions [5,8,15].

The study of exposure disparities has become increasingly complex due to changes in sources contributing to ambient PM_2.5_, driven by emissions regulations and climate change. In particular, wildfire smoke—a major contributor to ambient PM_2.5_—has increased in frequency, intensity, and geographic range due to climate change [16–18], changes in land management [19,20], and development in the wildland-urban interface [21], especially in the Western US and California. Since 2016, the increase in wildfire smoke PM_2.5_ has eroded about 25% of the policy-driven reductions in PM_2.5_ concentrations nationally and reversed nearly 50% of PM_2.5_ improvements in the Western US [22]. Wildfire smoke PM_2.5_ also demonstrates different spatial patterns than other sources of PM_2.5_. Unlike traditional sources of PM_2.5_ [23], wildfire PM_2.5_ disproportionately exposes white, Hispanic, American Indian, and more affluent populations in the US [24,25], as these populations tend to reside in areas where wildfires occur more frequently [26]. However, wildfire smoke poses a greater risk to disadvantaged groups, due to increased psychological stress [27] and limited resources to evacuate and prevent exposure (e.g., from residing in a well-built house that can effectively keep smoke out or from having the financial means to purchase air filters), and ultimately contributes to a widened climate gap [28]. Importantly, actions aimed at reducing wildfire smoke PM_2.5_ will significantly differ from those targeting traditional sources of PM_2.5_, primarily given that wildfire smoke cannot be as easily regulated through state and federal policies as traditional sources of PM_2.5_. Additionally, wildfires have multiple drivers, including climate change, wildland management practices, and development in the wildland-urban interface, complicating efforts to reduce exposure to wildfire smoke PM_2.5_. Considering how wildfire smoke affects the trends in PM_2.5_ exposure disparities can inform regulatory efforts to better prepare for a future with increasing wildfire smoke while promoting health equity.

California constitutes an ideal setting for studying time trends of disparities in PM_2.5_ exposure and the contribution from wildfire smoke PM_2.5_. California is a diverse state with relatively high ambient PM_2.5_ concentrations, of which wildfire PM_2.5_ emissions accounted for 66% of total PM_2.5_ emissions in 2015 [29] and are projected to increase in the future [30,31]. Ambient PM_2.5_ exposure disparities by SES and RE composition were previously observed in California [32].

This study examines the temporal changes in population-weighted and rank-ordered PM_2.5_ exposure at the census tract-level in California from 2006 to 2018 and whether community exposure disparities across multiple indicators of RE and SES change over time. We also explore the influence of wildfire smoke on the temporal changes and community disparities in PM_2.5_ exposure. Understanding how disparities in PM_2.5_ exposure and its wildfire-related component vary over time could provide insight into potential drivers of social inequities in health, highlight where action is needed to address such inequities, and support evidence-based policy development.

## Materials and methods

### Data sources

We utilized a previously developed time-series dataset [33], which provided daily total and wildfire PM_2.5_ concentrations, to calculate annual average total and wildfire PM_2.5_ concentrations at the census tract-level within California from 2006 to 2018. This dataset estimated daily total PM_2.5_ concentrations at the census tract-level with an ensemble model of multiple machine learning algorithms, measurement data from the US Environmental Protection Agency’s Air Quality System monitors, and a large set of predictor variables. Aguilera et al. identified wildfire smoke days through satellite smoke plume data and estimated census tract-level non-wildfire PM_2.5_ concentrations on those days through imputation with chained random forest algorithm and total PM_2.5_ during non-wildfire days. Wildfire PM_2.5_ equals total PM_2.5_ minus estimated non-wildfire PM_2.5._

To explore indicators for potential community disparities, we obtained yearly census tract-level age-specific population sizes and proportions of sociodemographic characteristics from the American Community Survey 5-year estimates for the study period, including indicators of RE (proportions of Non-Hispanic white, Black, Asian, American Indian/Alaska Native, Hawaiian Native and other Pacific Islander, and Hispanic, in accordance with the Office of Management and Budget Standards) [34], education (proportion of 25 years and older with a Bachelor’s degree or higher and proportion of 15–17 years enrolled in high school), employment (proportion of unemployment among 20–64 years), poverty (proportion of population with an income greater than 200% of the federal poverty level), and income (median annual household income) [35]. We assigned the 5-year estimates of 2006–2010 to years 2006–2010, the 5-year estimates of 2011–2015 to years 2011–2015, and the 5-year estimates of 2015–2019 to years 2016–2018. We used the 5-year estimates of 2015–2019 instead of the estimates of 2016–2020 to avoid influence from substantial changes in census tract boundaries in 2020. To facilitate calculations of population-weighted average exposure, we dichotomized the income variable into high (1) and low (0), where the boundary between the two is based on the year-specific median value of all census tracts in California. We coded SES indicators so that the disadvantaged groups are populations with unemployment, below poverty (below the federal poverty level), lower income, no college educational attainment, and no high school enrollment.

### Statistical analyses

Rank-rank comparisons were used to provide information on how PM_2.5_ exposure in each census tract changes over time relative to other census tracts in California. To provide an intuitive estimate of exposure that incorporates population distribution, we also calculated annual- and study period-average population-weighted PM_2.5_ concentrations across census tracts in California. We conducted analyses for total, wildfire, and non-wildfire PM_2.5_ concentrations separately.

First, we evaluated temporal changes in rank-ordered exposure to annual average PM_2.5_ concentrations across census tracts from 2006 to 2018 with rank-rank comparisons, focusing on a comparison of the starting and ending years of our study period. The rank-rank comparison orders census tracts from 2006 and 2018 in increasing PM_2.5_ rank and compares the percentile rank of the former period to the mean percentile rank of the latter period. The rank-rank comparison demonstrates whether census tracts historically exposed to the highest levels of pollution remained the most exposed in later years. We calculated Spearman’s correlation coefficient to assess the strength and direction of the correlation between the two periods, which indicates how PM_2.5_ exposure in each census tract has changed over time. As a sensitivity analysis, we also assessed the rank-rank comparisons for annual average PM_2.5_ concentrations between 2006 and all years from 2007 to 2018, providing the full picture of relative change across the study period.

Next, we used annual population-weighted average PM_2.5_ concentrations for total and sociodemographic populations to evaluate temporal changes and heterogeneity in PM_2.5_ exposure across subgroups, as well as potential temporal changes in such heterogeneity [36]. We used the total or subgroup-specific yearly population size as the weight for each census tract. We also calculated the absolute differences in population-weighted average PM_2.5_ concentrations across subgroups for each sociodemographic indicator. For RE indicators, differences were calculated as the RE group average minus the non-RE group average. For socioeconomic indicators, differences were calculated as the disadvantaged group average minus the advantaged group average. To provide insights into the community characteristics, we summarized the average sociodemographic indicators among the census tracts exposed to the lowest and highest 10% of three types of PM_2.5_ concentrations in 2006–2008 and 2016–2018. We chose the average PM_2.5_ concentrations of three years so that we identified the lowest and highest 10% exposed communities using the same length of period used by the US Environmental Protection Agency in air pollution policy compliance evaluations. The analysis was done with R version 4.1.0 [37].

### Ethics statement

Ethical approval was not required as this study did not involve animals and human subjects. We accessed the American Community Survey 5-year estimates on March 7, 2024, and we have no access to information that could identify individual participants.

## Results

This study spanned 2006–2018 and focused on 7,594 (94.2%) California census tracts with complete data for PM_2.5_ exposure and sociodemographic indicators. We removed 1 (<0.1%) census tract due to missing PM_2.5_ exposure data, 239 (3.0%) census tracts due to population sizes less than 1,500, and 224 (2.8%) census tracts due to lack of sociodemographic indicator data. The 2006–2018 population-weighted average total, wildfire, and non-wildfire PM_2.5_ concentrations across all included California census tracts were 10.70 μg/m^3^, 0.26 μg/m^3^, and 10.44 μg/m^3^, respectively.

### Temporal change in total PM_2.5_

**Fig 1.**
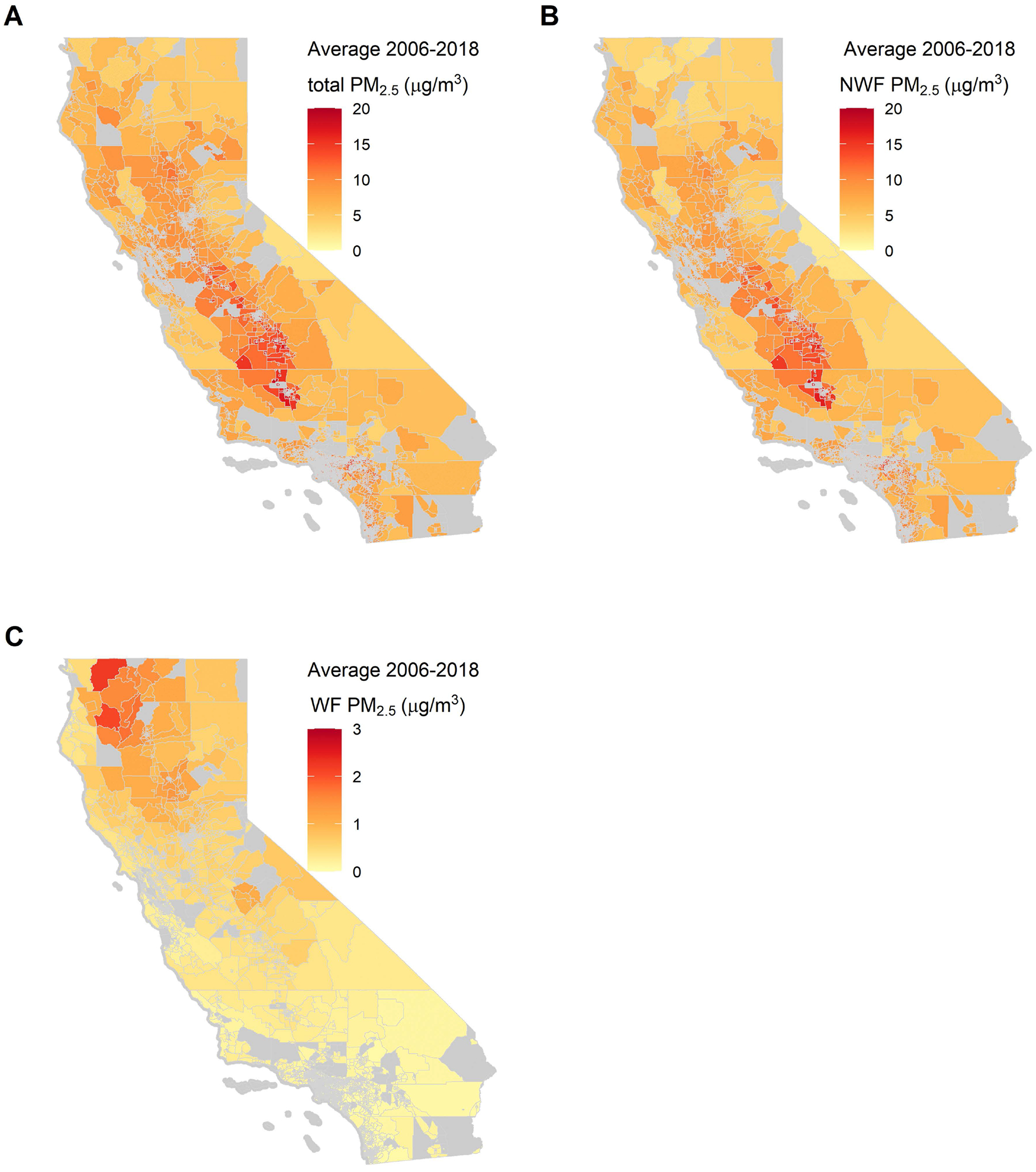
Spatial distribution of census tract-specific annual average concentration. The panels are census tract-specific annual average concentrations between 2006–2018: A) total mass PM_2.5_; B) non-wildfire (NWF) PM_2.5_; and C) wildfire (WF) PM_2.5_. Gray areas were census tracts excluded due to missing data and population sizes smaller than 1,500. This figure was created using publicly available 2010 US Census TIGER/Line Shapefiles, provided by the US Census Bureau at: https://www.census.gov/geographies/mapping-files/time-series/geo/tiger-line-file.2010.html#list-tab-790442341. https://doi.org/10.1371/journal.pclm.0000796.g001

Across all included California census tracts, the median annual average total PM_2.5_ concentration decreased with some fluctuations over time (S1A Fig) and heterogeneity across space (Fig 1A). In taking the temporal trend of population composition into consideration, the state-level population-weighted average total PM_2.5_ concentration demonstrated similar fluctuations as the median annual average across California census tracts, which decreased from 11.85 μg/m^3^ in 2006 to 10.92 μg/m^3^ in 2018, a 7.9% reduction (S1 Table). Census tracts with larger decreases in annual average total PM_2.5_ concentrations from 2006 to 2018 clustered in the Los Angeles metropolitan area and San Joaquin Valley, while census tracts with larger increases in total PM_2.5_ were in the Mountain Counties and Sacramento Valley (S2A Fig). Areas with larger decreases overlapped with areas with higher total PM_2.5_ concentrations in 2006 (S3A Fig).

**Fig 2.**
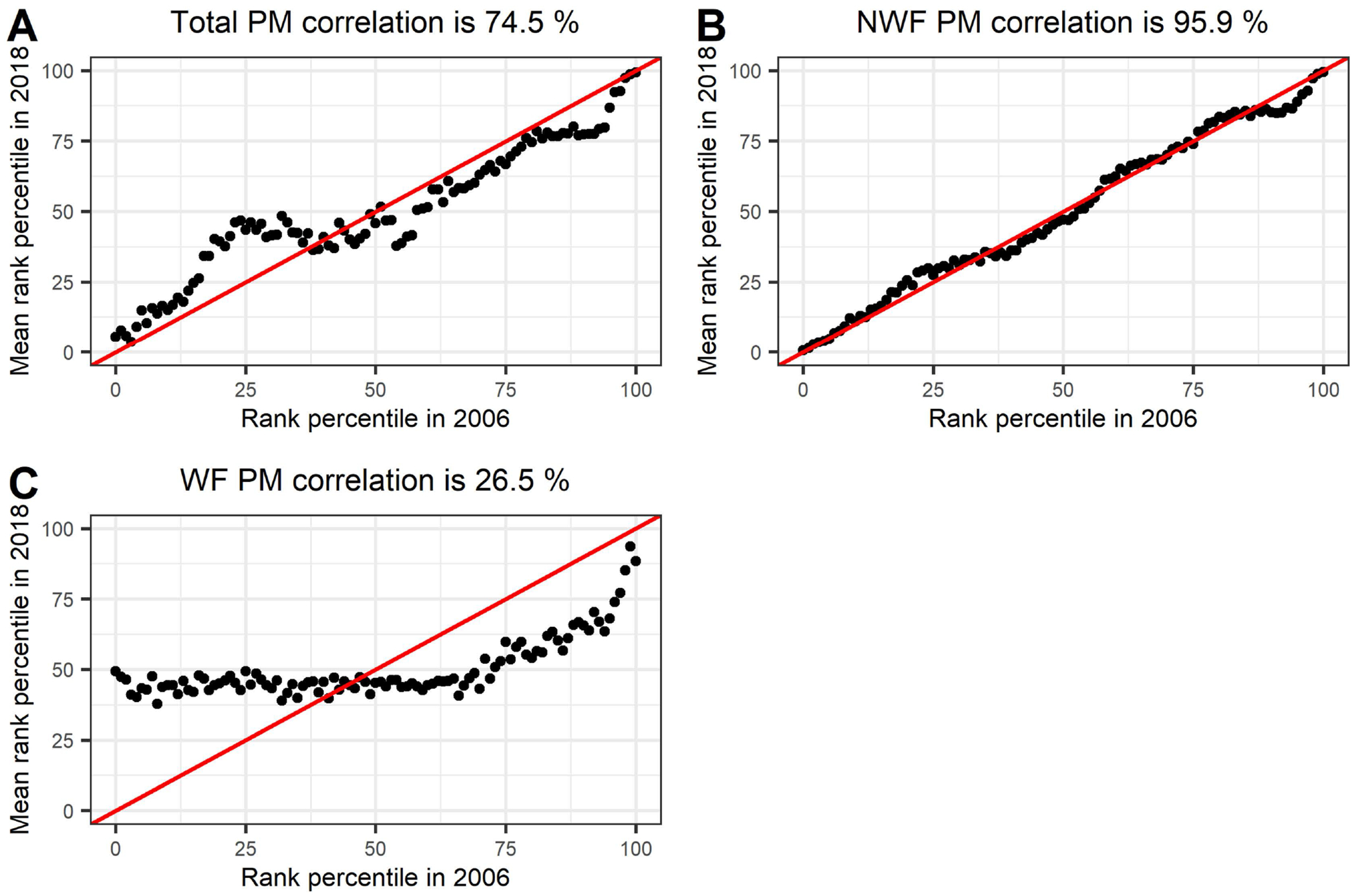
Rank-rank comparisons of PM_2.5_ concentrations between 2006 and 2018. The panels are: A) total PM_2.5_; B) non-wildfire PM_2.5_; and C) wildfire PM_2.5_. The red line is the 45° line. https://doi.org/10.1371/journal.pclm.0000796.g002

The rank order of total PM_2.5_ exposure across most census tracts remained stable from 2006 compared pairwise to all subsequent years (Fig 2A, S4A Fig – S14A Fig in S1 File), suggesting that census tracts historically most and least exposed continued to remain most and least exposed, respectively. However, census tracts historically less exposed in 2006 (around 25^th^ percentile) became slightly more exposed in 2018 (Fig 2A), and those historically more exposed in 2006 (around 90^th^ percentile) became less exposed in 2010–2018 (Fig 2A, S7A Fig – S14A Fig in S1 File). The Spearman’s correlation coefficients were high 90% across all comparisons except for the 2006–2018 comparison, which was 74.5%.

### Temporal change in non-wildfire PM_2.5_

Similar to total PM_2.5_, the median annual average non-wildfire PM_2.5_ concentration decreased from 2006 to 2018 (S1B Fig). In taking the temporal trend of population composition into consideration, the state-level population-weighted average non-wildfire PM_2.5_ concentration decreased from 11.75 μg/m^3^ in 2006 to 9.78 μg/m^3^ in 2018, a 16.8% reduction (S1 Table). Most census tracts demonstrated decreasing annual average non-wildfire PM_2.5_ concentrations from 2006 to 2018, with larger absolute decreases clustered in the San Joaquin Valley and Los Angeles metropolitan area (S2B Fig), similar to areas with higher reduction in total PM_2.5_ concentrations.

From 2006 to 2018, we observed no temporal changes in the rank-ordered non-wildfire PM_2.5_ concentrations except for slight decreases in ranks among those most exposed, with a Spearman’s correlation coefficient of 95.9% (Fig 2B). The slight decreases in ranks among those most exposed were present in comparisons of 2006 to years after 2009 (S7B Fig – S14B Fig in S1 File).

### Temporal change in wildfire PM_2.5_

Compared to total PM_2.5_ and non-wildfire PM_2.5_, the median census tract wildfire PM_2.5_ concentration fluctuated more over time, with a slight increasing trend in recent years and substantially higher concentrations in 2008 and 2018 compared to 2006 (S1C Fig). The state-level population-weighted average wildfire PM_2.5_ concentration demonstrated similar fluctuation as the median annual average across California census tracts, with the lowest value in 2011 at 0.03 μg/m^3^, and the highest value in 2018 at 1.14 μg/m^3^ (S1 Table). When comparing the year of 2018 with an exceptionally high population-weighted average wildfire PM_2.5_, to the year of 2006 with relatively low wildfire PM_2.5_ at 0.12 μg/m^3^, we observed increases in annual average wildfire PM_2.5_ concentration in most census tracts, with particularly large increases in census tracts in the Mountain Counties and Sacramento Valley (S2C Fig). These areas overlapped with areas with larger increases in total PM_2.5_ (S2A Fig) and areas less exposed to non-wildfire PM_2.5_ (S3C Fig, S3D Fig).

Over time, census tracts most exposed to wildfire PM_2.5_ in 2006 became less exposed in 2018, while census tracts least exposed became more exposed, indicated by a Spearman’s correlation coefficient of 26.5% (Fig 2C). Similar observations were found in comparisons of 2006 to other years as well (S4C Fig – S14C Fig in S1 File). However, this temporal change in rank-ordered wildfire PM_2.5_ exposure only visibly influenced the temporal change in rank-ordered total PM_2.5_ during 2018, potentially due to the high concentrations of wildfire PM_2.5_ in 2018 (1.14 μg/m^3^ compared to < 0.70 μg/m^3^ in other years). If the temporal change in rank-ordered wildfire PM_2.5_ exposure in 2018 were to be ignored or removed, the amplitude of the temporal change in rank-ordered total PM_2.5_ would be diminished.

### Disparity across socioeconomic indicators and changes over time

In both the 2006–2008 and 2016–2018 periods, census tracts most exposed to total PM_2.5_ (≥90^th^ percentile for total PM_2.5_) had higher proportions of populations who were impoverished or had no college educational attainment, compared to those least exposed (≤10^th^ percentile in total PM_2.5_) (S2 Table). Similarly, census tracts most exposed to non-wildfire PM_2.5_ had higher proportions of populations who were impoverished or had no college educational attainment, compared to those least exposed (S2 Table). Census tracts most versus least exposed to wildfire PM_2.5_ had higher proportions of populations who were impoverished, unemployed, and had no college educational attainment, but to a lesser extent than for total and non-wildfire PM_2.5_ (S2 Table).

**Fig 3.**
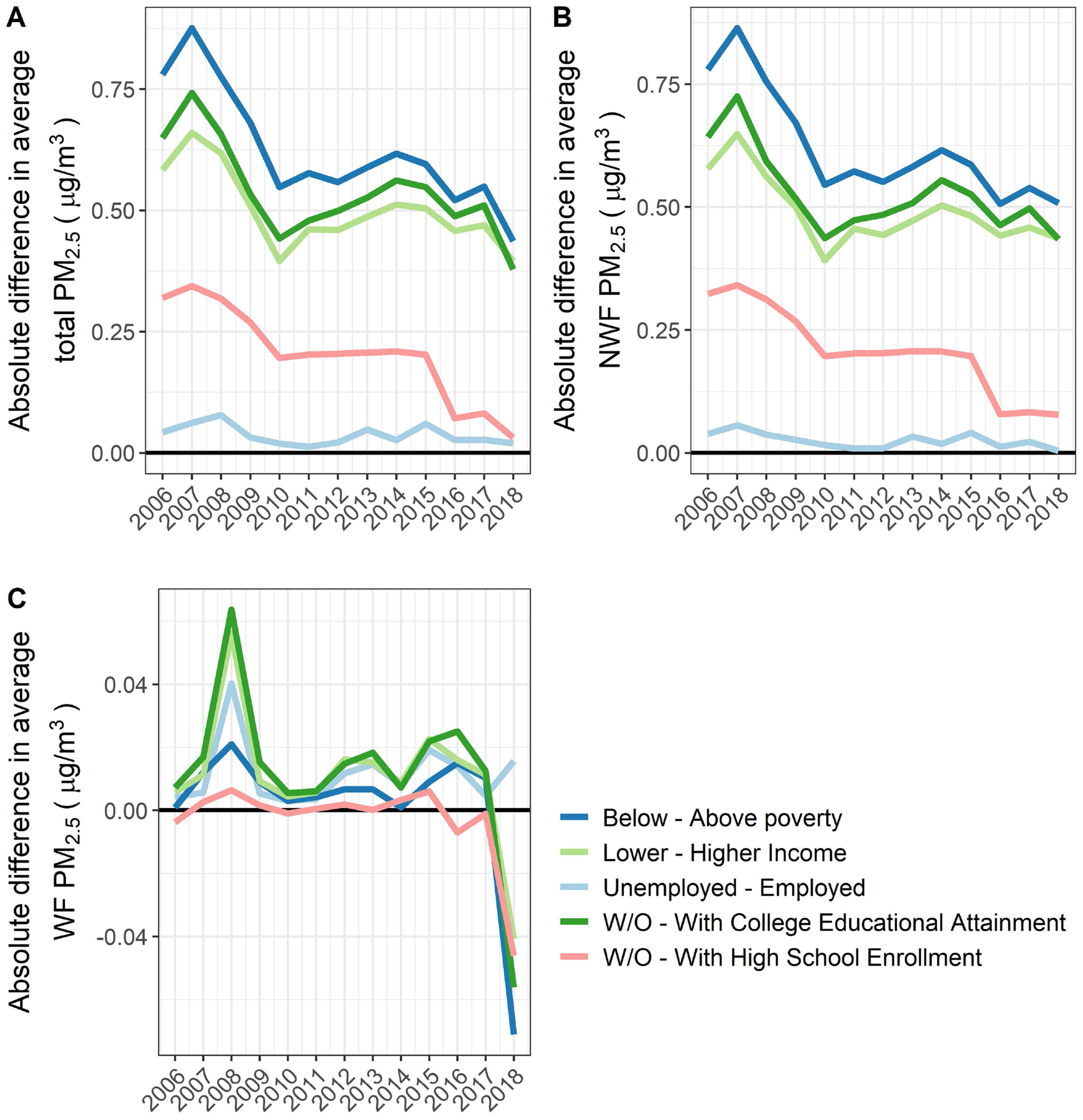
Differences in the population-weighted average PM_2.5_ concentrations between socioeconomic groups. Differences in the population-weighted average PM_2.5_ concentrations between socioeconomic groups calculated as the disadvantaged group average minus advantaged group average across the study period for: A) total PM_2.5_; B) non-wildfire PM_2.5_; and C) wildfire PM_2.5_. The disadvantaged groups are: populations with an income that is lower than 200% of the federal poverty level, populations with median annual household income lower than the state median, 20–64 years unemployed populations, 25 years and older without a Bachelor’s degree, and 15–17 years not enrolled in high school. https://doi.org/10.1371/journal.pclm.0000796.g003

To assess disparities across socioeconomic indicators and their changes over time, we calculated annual population-weighted average PM_2.5_ concentrations among different SES groups. Absolute differences in total PM_2.5_ were positive for all disadvantaged groups in each year of the study period, indicating that disadvantaged groups (i.e., unemployed, impoverished, low income, no college educational attainment, and no high school enrollment groups) consistently experienced higher exposure, compared to their counterparts (Fig 3A). Absolute differences between disadvantaged and advantaged groups were larger for indicators of poverty, low income, and no college educational attainment, compared to indicators of unemployment and no high school enrollment (Fig 3A). Absolute differences shrank during 2007–2010 and 2014–2018 and increased during 2010–2014 for all groups, which were especially prominent for indicators of poverty, low income, low college educational attainment and low high school enrollment. The overall trend of narrowed, but persistent, absolute differences imply reductions in community exposure disparities over time.

The trends of absolute differences in non-wildfire PM_2.5_ over time mirrored those of total PM_2.5_ (Fig 3B), with positive differences suggesting higher exposure for disadvantaged groups within every year. Similar to total PM_2.5_, absolute differences in non-wildfire PM_2.5_ shrank during 2007–2010 and 2014–2018 and increased during 2010–2014 (Fig 3B). In contrast, the trends of absolute differences in wildfire PM_2.5_ over time largely differed from the trends in total PM_2.5_ and non-wildfire PM_2.5_, with near-zero but generally positive differences across SES groups in each year, aside from 2008 and 2018 (Fig 3C, S1 Table). In 2008, we observed positive differences, indicating exaggerated community exposure disparities (Fig 3C). Wildfire PM_2.5_ contributed to 51.8%, 2.7%, 9.7%, 2.0%, and 9.1% of the community exposure disparities in total PM_2.5_ in 2008 for unemployment, poverty, college educational attainment, high school enrollment, and income, respectively (calculated as the difference in average wildfire PM_2.5_ between subgroups divided by the difference in average total PM_2.5_ between subgroups). In 2018, differences in wildfire PM_2.5_ steeply decreased to negative values for all indicators except for unemployment (Fig 3C), implying that all advantaged groups, except for employed groups, experienced higher exposure. Thus, in 2018, wildfire PM_2.5_ contributed to decreases of 14.0%, 12.9%, 59.5%, and 9.4% from the community exposure disparities in non-wildfire PM_2.5_ for poverty, college educational attainment, high school enrollment, and income, respectively (calculated as the average wildfire PM_2.5_ between subgroups divided by the difference in average non-wildfire PM_2.5_ between subgroups).

In summary, community exposure disparities in California for total and non-wildfire PM_2.5_ rapidly narrowed during 2007–2010. Community exposure disparities for wildfire PM_2.5_ varied over time, where disadvantaged groups experienced higher exposure during 2006–2017, with particularly notable increased disparities in 2008, while advantaged groups experienced higher exposure in 2018.

### Disparity across race/ethnicity and changes over time

In both the 2006–2008 and 2016–2018 periods, census tracts most exposed to total PM_2.5_ (≥90^th^ percentile in total PM_2.5_) had higher proportions of Black, Asian, and Hispanic populations, compared to those least exposed (≤10^th^ percentile in total PM_2.5_) (S2 Table). Similarly, the census tracts most exposed to non-wildfire PM_2.5_ had higher proportions of Black, Asian, and Hispanic populations, compared to those least exposed (S2 Table). Contrary to total and non-wildfire PM_2.5,_ census tracts with a larger proportion of white populations were more exposed to wildfire PM_2.5_.

**Fig 4.**
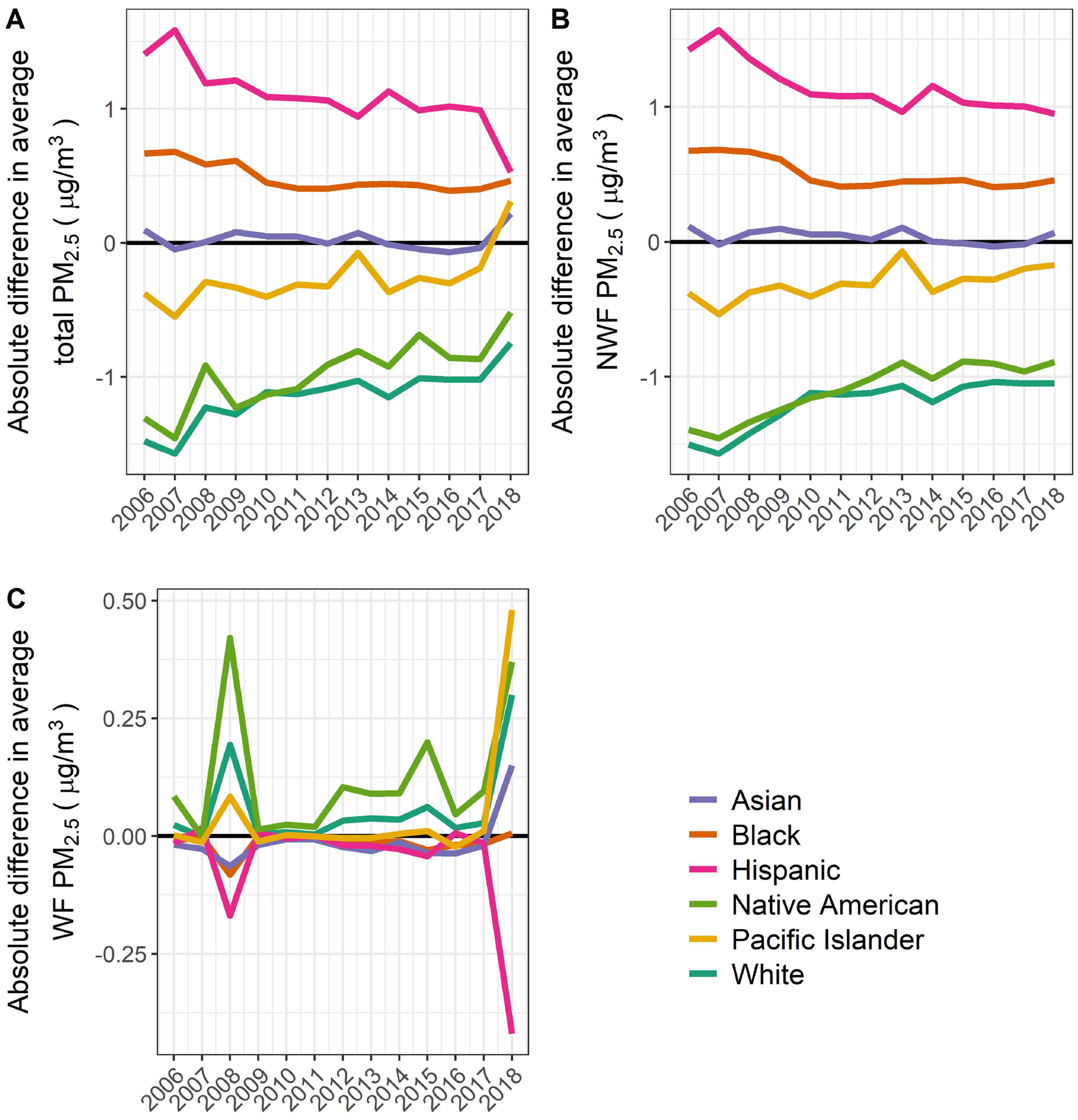
Differences in the population-weighted average PM_2.5_ concentrations between race and ethnicity groups. Differences in the population-weighted average PM_2.5_ concentrations between race and ethnicity groups calculated as the race and ethnicity group average minus the non-race and ethnicity group average (e.g., the population-weighted average among Asian population minus the population-weighted average among non-Asian population) across the study period for: A) total PM_2.5_; B) non-wildfire PM_2.5_; and C) wildfire PM_2.5_. https://doi.org/10.1371/journal.pclm.0000796.g004

To assess disparities across RE indicators and their changes over time, we calculated annual population-weighted average PM_2.5_ concentrations among RE groups of non-Hispanic white, Black, Asian, American Indian or Alaska Native, Hawaiian Native and other Pacific Islander, and Hispanic populations (hereon referred to as white, Black, Asian, Native American, Pacific Islander, and Hispanic populations, respectively). Absolute differences in total PM_2.5_ were mostly positive for Black and Hispanic populations and mostly negative for white, Native American, and Pacific Islander populations (Fig 4A). For Asian populations, the differences fluctuated around zero (Fig 4A). The observed positive differences suggest that Black and Hispanic populations were exposed to higher PM_2.5_ than non-Black and non-Hispanic populations, while the observed negative differences suggest that white, Native American, and Pacific Islander populations were exposed to lower PM_2.5_ than non-white, non-Native American, and non-Pacific Islander populations. Absolute differences shrank from 2006 to 2018 for all groups, with a notably large shrinkage in 2018 for all groups aside from Asian and Pacific Islander populations, who experienced higher exposure to PM_2.5_ than non-Asian and non-Pacific Islander populations. These results suggest that over the study period, community exposure disparities for total PM_2.5_ have reduced for white, Black, Native American, and Hispanic populations, compared to their counterparts.

The trends of absolute differences in non-wildfire PM_2.5_ over time were similar to the trends in total PM_2.5_, with mostly positive differences for Hispanic and Black populations, mostly negative differences for white, Native American, and Pacific Islander populations, and near-zero differences for Asian populations (Fig 4B). Similar to total PM_2.5_, absolute differences in non-wildfire PM_2.5_ shrank towards zero from 2006 to 2018, but the shrinkage progressed more slowly beginning in 2010 for all groups aside from Black and Asian populations (Fig 4B). However, absolute differences in non-wildfire PM_2.5_ remained fairly constant for all groups in 2018, compared to absolute differences in total PM_2.5_, which notably shrank for Hispanic, Native American, and white populations and widened for Asian and Pacific Islander populations in 2018 (Fig 4B).

Conversely, the trends of absolute differences in wildfire PM_2.5_ over time were strikingly distinct from the trends in total PM_2.5_ and non-wildfire PM_2.5_, with mostly positive differences for Native American and white populations and fluctuating near-zero differences for all other groups (Fig 4C). Larger absolute differences in wildfire PM_2.5_ were apparent in 2008 and 2018, compared to other years (Fig 4C). In 2018, the pattern of differences suggested increased exposure to wildfire PM_2.5_ for Asian, Native American, white, and Pacific Islander populations and decreased exposure for Hispanic populations, with changes in wildfire PM_2.5_ representing 44.3% of the decreases in disparity of total PM_2.5_ for Hispanic and non-Hispanic populations. Since Native American and white populations started with lower total PM_2.5_ than their counterparts, increases in their wildfire exposure PM_2.5_ contributed to reductions in disparity.

In summary, community exposure disparities for non-wildfire PM_2.5_ narrowed over time from 2006 to 2018. During years with high wildfire activity, such as in 2008 and 2018, wildfire PM_2.5_ was higher among white, Native American, and Pacific Islander populations, and lower among Hispanic populations, showing disparities favoring Hispanic populations.

## Discussion

Previous studies in the US have documented a reduction in absolute exposure disparities to total PM_2.5_ (as a marker of overall atmospheric pollution) among RE and SES groups since 2000 [5,6,8,13]. However, wildfire smoke has become a main contributor to PM_2.5_ concentrations in a changing climate [30]. Compared to traditional sources of PM_2.5_ like traffic or industry, wildfires tend to occur in less predictable locations, resulting in a more random spatial distribution of wildfire PM_2.5_ exposure, which does not necessarily coincide with the spatial distribution of exposure from traditional pollution sources. These spatial variations underscore the stark contrast in exposure patterns between the two pollution sources, reinforcing the need to account for and address disparities in wildfire smoke exposure. In this California-based study spanning 2006–2018, we find reduced, but persistent, community exposure disparities for total PM_2.5_. However, these reduced disparities across SES and RE indicators, particularly the decrease in 2018, a year with high wildfire PM_2.5_ exposure, were 9.4% to 59.5% attributable to increased wildfire PM_2.5_ exposure among those historically less impacted by traditional sources of PM_2.5_. Our finding indicates that substantial efforts are still needed to address inequities in air pollution exposure related to traditional sources of PM_2.5_.

Specifically, we found that, between 2006 and 2018 in California, total PM_2.5_ concentrations decreased as wildfire PM_2.5_ concentrations fluctuated with peaks in 2008 and 2018. We observed a reordering of rank-ordered exposure to total PM_2.5_ from 2006 to 2018, but the amplitude of these changes diminished when excluding the wildfire PM_2.5_ contribution and focusing on non-wildfire PM_2.5_ alone. We also observed a reduction in community exposure disparities in total PM_2.5_ across RE and SES indicators, with the large decrease in 2018 driven mostly by increased wildfire PM_2.5_ exposure among less disadvantaged groups. Thus, in the absence of wildfire PM_2.5_ contributions to the trends in total PM_2.5_ exposure changes and disparities, improvements towards exposure equity were less pronounced than they appeared in 2018.

The observed decreases in absolute total and non-wildfire PM_2.5_ concentrations over time, alongside persistent SES and RE disparities align with the current literature [5,8,15]. The promulgation of air pollution control policies that target anthropogenic sources of PM_2.5_, such as the National Ambient Air Quality Standards for PM_2.5_, supported some reductions in exposure disparities across subpopulations. However, our results show that despite these policies, disparities persist, as demonstrated by the observed positive absolute differences in total PM_2.5_ across SES and RE groups. The observed community exposure disparities reflect continued institutional and systemic racism, as in the case with health disparities [38]. For instance, redlining, a historical practice that facilitated housing segregation and discriminated against minoritized and low-income populations, has persistent effects including racial segregation, wealth accumulation, and industrial exposures that continue to influence air pollution exposure today [10]. To better achieve disparity elimination, a previous study has suggested policies that specifically focus on reducing exposure disparities [39].

Importantly, wildfire PM_2.5_ partially explained the observed temporal change in rank-ordered exposure to total PM_2.5_ in California since 2006, particularly in 2018, a year with high wildfire frequency and intensity. Communities most exposed to non-wildfire PM_2.5_ in the past continue to face the highest exposures years later—an observation obscured by wildfire PM_2.5_ when evaluating rank-order exposure to total PM_2.5_ between 2006 and 2018. In other words, wildfire PM_2.5_ has exaggerated the progress in reducing inequities in traditional sources of PM_2.5_. This has implications for groups who are forced to continue to bear the health burdens resulting from inequitable total PM_2.5_ exposure. More targeted efforts in reducing traditional sources of PM_2.5_ are needed to protect these groups.

Our results also suggest that a portion of the reduction in community exposure disparities for total PM_2.5_ in California were attributable to higher wildfire PM_2.5_ exposure among those historically less impacted by traditional sources of PM_2.5_ in 2018 (e.g., accounting for 44.3% of the decreases in total PM_2.5_ disparities between Hispanic and non-Hispanic populations and 59.5% of the decreases in total PM_2.5_ disparities between populations with and without high school enrollment), rather than regulatory efforts to reduce traditional sources of PM_2.5_. However, reductions in total PM_2.5_ disparities that originate from increased exposure to wildfire PM_2.5_ among historically less impacted groups still contribute to the total health burden. Our results support development of air pollution control policies that explicitly target eliminating disparities across subpopulations historically exposed to high concentrations of PM_2.5_, especially non-wildfire PM_2.5_. Such strategies include location-specific interventions based on observed disparities in exposure and susceptibility [40]. Although racially and socioeconomically marginalized communities are generally exposed to less wildfire PM_2.5_ than their counterparts in years like 2018, these communities face higher health impacts from these hazards, due to limited resources to mitigate exposure, psychological stress, and higher prevalence of pre-existing conditions, partly driven by persistent structural racism [26,27,41]. Therefore, wildfire smoke exposure reduction methods, such as improved forest management, wildfire smoke warnings, establishment of clean air center, and viable self-protection methods, should be promoted across all populations.

We found the contribution of wildfire PM_2.5_ to the reduction of total PM_2.5_ disparities was consistent across RE groups for the entire study period, but this varied temporally by SES group (i.e., exaggerated disparities in 2008 vs. reduced disparities in 2018). Most wildfires occur in rural areas, where communities tend to be comprised of larger proportions of white populations, as supported by the mean RE indicators among the least and most exposed census tracts (S2 Table). The high wildfire PM_2.5_ exposures in these areas diminished typical disparities in PM_2.5_ exposure across RE groups [26]. On the other hand, the SES indicators among the least and most wildfire-exposed census tracts were smaller, suggesting a more homogeneous distribution of wildfires across SES indicators, which can explain the observed fluctuations in the influence of wildfires on disparity across SES groups over time. This pattern also reveals the unpredictable and random nature of wildfire smoke and highlights the importance of developing targeted efforts in reducing traditional sources of PM_2.5_.

This study has several limitations. First, we demonstrated the exposure disparities across sociodemographic indicators using population-weighted averages, but we were not able to simultaneously evaluate the disparity across multiple indicators or their interactions (e.g., communities both racially and socioeconomically marginalized might experience higher exposure disparities than communities experiencing just one aspect of marginalization). Second, we used census tract-level average ambient PM_2.5_ to represent the average population exposure for the census tract; however, individuals of marginalized groups might experience higher exposure than others within the same census tract, due to siting of point sources of pollution and locations of roads resulting in traffic-related air pollution, which may not be captured in our analysis. Third, we only focused on wildfire smoke as a specific source of ambient PM_2.5_. Some studies have integrated other sources of ambient PM_2.5_ to provide more insights into exposure disparity trends in the US [14], but future studies should evaluate these trends at a finer spatial resolution and in California specifically.

Future research could consider additional indicators that this study did not analyze, such as adaptive capacity variables and variables measuring structural racism (e.g., residential segregation and index of disproportionality), when exploring disparities in PM_2.5_ exposure [42,43]. It could also be informative to investigate intersectionality among these sociodemographic indicators and identify communities with a combination of these indicators that have experienced the highest disparities and improvements over time [4]. Lastly, future studies could explore whether disparities in exposure to wildfire-related air pollutants outside of PM_2.5_ exist, such as ozone or polycyclic aromatic hydrocarbons.

In sum, our findings suggest that though total PM_2.5_ concentrations have decreased from 2006 to 2018 in California, wildfire PM_2.5_ concentrations fluctuated with large increases in certain years. Furthermore, we showed that exposure disparity to total PM_2.5_ decreased but persisted across space and indicators of RE and SES. Communities historically ranked as highly exposed to total PM_2.5_ became less exposed in recent years and the gap in exposure to total PM_2.5_ narrowed across RE and SES groups. However, such reductions were partially attributed to wildfires and their disproportionate impacts on advantaged communities, rather than from policies aimed at improving air quality in disadvantaged communities. These findings indicate that targeted efforts are still needed to address the existing PM_2.5_ disparities contributing to environmental injustice in California.

## Supplementary Material

S1_Fig S1 Fig. Boxplot of annual average total, wildfire, and non-wildfire PM_2.5_ concentration across the study period in California (μg/m^3^): A) annual average total PM_2.5_; B) annual average non-wildfire PM_2.5_; and C) annual average wildfire PM_2.5_.

S1_Table **S1 Table. Differences in the population-weighted average PM_2.5_ concentrations between race and ethnicity groups and socioeconomic groups.** Numerical results for Figure 3 and 4.

S2_Table S2 Table. Average sociodemographic indicators among census tracts least (≤10^th^ percentile) and most exposed (≥90^th^ percentile) to total, non-wildfire (NWF) and wildfire (WF) PM_2.5_ in averages of years 2006–2008 and averages of years 2016–2018.

S2_Fig **S2 Fig. Spatial distribution of census tract-specific annual average concentrations differences of the year 2018 minus the year 2006 for: A) total mass PM_2.5_; B) non-wildfire (NWF) PM_2.5_; and C) wildfire (WF) PM_2.5_.** Gray areas were census tracts excluded due to missing data and population sizes smaller than 1,500. This figure was created using publicly available 2010 US Census TIGER/Line Shapefiles, provided by the US Census Bureau at: https://www.census.gov/geographies/mapping-files/time-series/geo/tiger-line-file.2010.html#list-tab-790442341.

S3_Fig **S3 Fig. Spatial distribution of average wildfire and non-wildfire PM_2.5_ concentration in California (μg/m^3^): A) annual average total PM_2.5_ in 2006; B) annual average total PM_2.5_ in 2018; C) annual average non-wildfire PM_2.5_ in 2006; D) annual average non-wildfire PM_2.5_ in 2018; E) annual average wildfire PM_2.5_ in 2006; and F) annual average wildfire PM_2.5_ in 2018.** Gray areas were excluded census tracts due to missing data. This figure was created using publicly available 2010 US Census TIGER/Line Shapefiles, provided by the US Census Bureau at: https://www.census.gov/geog-raphies/mapping-files/time-series/geo/tiger-line-file.2010.html#list-tab-790442341.

S1_File **S1 File. S4 Fig - S14 Fig. Rank-rank comparisons of PM_2.5_ concentrations between 2006 and all years between 2007 and 2017: A) total PM_2.5_; B) non-wildfire PM_2.5_; and C) wildfire PM_2.5_.** The red line is the 45° line.

## Data Availability

All data and codes are available at https://github.com/benmarhnia-lab/cal_pm25_disparity_wildfire.
