# Supplementary material for: The diverging role of increasing wildfire smoke to ambient PM2.5 exposure disparity in California, 2006 to 2018
Source: PLOS Clim. Author manuscript; Available in PMC 2026 Jun 18. (PMC13274699; doi:10.1371/journal.pclm.0000796)
Supplement: S1_Table — S1 Table. Differences in the population-weighted average PM2.5 concentrations between race and ethnicity groups and socioeconomic groups. Numerical results for Figure 3 and 4. [file NIHMS2173162-supplement-S1_Table.pdf]

Exposure	Year	Average Concentration ($\mu g/m^3$)	Population-Weighted Average Concentration ($\mu g/m^3$)	Population-Weighted Average Concentrations for Socioeconomic Groups ($\mu g/m^3$)									
				Employed	Unemployed	Above Poverty	Below Poverty	With College Educational Attainment	Without College Educational Attainment	With High School Enrollment	Without High School Enrollment	Higher Income	Lower Income
Total $PM_{2.5}$	2006	11.84554938	11.87610996	11.81395902	11.85659103	11.60052428	12.38069705	11.31753234	11.96679451	12.01984921	12.33958375	11.29546878	11.87906817
	2007	12.23178879	12.26678089	12.18824007	12.25001766	11.95730378	12.83342195	11.62827857	12.37034006	12.43927211	12.78321068	11.61031786	12.27012539
	2008	12.2920832	12.31060832	12.23817843	12.31592182	12.03657035	12.81236162	11.75584456	12.41269312	12.43329326	12.75163413	11.69569196	12.31374115
	2009	11.0692695	11.09106068	11.04234116	11.07420781	10.85062514	11.53128919	10.63282128	11.1656267	11.20231548	11.47144707	10.58425406	11.09364272
	2010	10.0319428	10.06108785	10.02163241	10.04055496	9.867571055	10.41540989	9.677009629	10.11860978	10.16692891	10.36304355	9.66736086	10.06309379
	2011	10.90268708	10.9267755	10.90796842	10.92031447	10.71701802	11.29379398	10.5241477	11.0031881	11.03954702	11.24220629	10.46791653	10.92912592
	2012	10.05123229	10.06929151	10.0510889	10.07237425	9.866339529	10.42440221	9.658658546	10.15753464	10.17285561	10.37749256	9.612363253	10.07163204
	2013	10.47209425	10.5133171	10.48004558	10.52780088	10.29925827	10.88786177	10.07542893	10.60179826	10.61746442	10.8242501	10.02887577	10.51579857
	2014	10.18020372	10.20015641	10.17799604	10.20455228	9.975883855	10.5925723	9.740145673	10.30206594	10.31667251	10.52599434	9.691000533	10.20276447
	2015	9.899838569	9.917665606	9.88045843	9.940120249	9.701258494	10.29631913	9.468685851	10.01649844	10.02945015	10.23230978	9.415877285	9.920235928
	2016	9.387565776	9.394110181	9.397589576	9.423794666	9.231730425	9.752621672	9.015128699	9.5033462	9.478997536	9.550379506	8.939026427	9.39647103
	2017	9.566080812	9.592342471	9.591740753	9.618666684	9.421147972	9.970315664	9.190951452	9.700886686	9.682439514	9.764119867	9.125207881	9.594765837
	2018	10.92432475	10.91805514	10.92396811	10.9439248	10.78192525	11.21861066	10.62642298	11.00482614	10.9676537	10.99897996	10.52489141	10.92009477
NWF $PM_{2.5}$	2006	11.72079004	11.75230944	11.69222879	11.73074072	11.47702758	12.25634026	11.19864916	11.84072968	11.8953822	12.21891152	11.177355	11.75523867
	2007	11.96632606	11.99836103	11.92372905	11.97985103	11.69312606	12.55723486	11.37455935	12.09971475	12.16595936	12.50723786	11.35315176	12.00164819
	2008	11.5855024	11.61210793	11.55739125	11.5948425	11.34548365	12.10028702	11.10049967	11.69363283	11.7367333	12.04863434	11.05299266	11.61495647
	2009	10.93600226	10.95701618	10.91155179	10.93803152	10.71975548	11.39143166	10.51100735	11.0285341	11.06392882	11.33147345	10.45945056	10.95955114
	2010	10.00372541	10.03238091	9.994452604	10.01038745	9.839903614	10.38479966	9.652523852	10.08863575	10.13742481	10.33449116	9.642969754	10.03436485
	2011	10.87564585	10.89945958	10.88250919	10.89120525	10.69123369	11.26379818	10.50145853	10.97444621	11.01134042	11.21354729	10.44620683	10.90178128
	2012	9.986205743	10.00811446	9.99481417	10.00439254	9.807599844	10.35896045	9.607076707	10.09119439	10.11242721	10.31522354	9.567235424	10.01037279
	2013	10.31326907	10.35525601	10.32868657	10.36180007	10.14362348	10.72555533	9.930382391	10.43843704	10.45677529	10.6634331	9.885922875	10.35766009
	2014	10.10201787	10.12547658	10.10643847	10.12489646	9.901546236	10.51729371	9.669162845	10.22389608	10.2436367	10.44969131	9.625021856	10.12804007
	2015	9.757180707	9.778637228	9.74990092	9.790414798	9.565527001	10.1515221	9.344289656	9.870169778	9.890865503	10.08771998	9.299376474	9.781092157
	2016	9.175843972	9.17915574	9.189902373	9.202009516	9.021432137	9.527387104	8.819543934	9.282769147	9.256460889	9.334833675	8.739953205	9.181434201
	2017	9.267061474	9.293596755	9.296769898	9.319069943	9.125617122	9.664471979	8.901537876	9.398984623	9.383295696	9.466086518	8.837726591	9.295961684
	2018	9.780904406	9.779501897	9.794549077	9.798877961	9.621213389	10.12898049	9.441023165	9.875616416	9.84991341	9.927293435	9.345778094	9.781751937
WF $PM_{2.5}$	2006	0.12475929	0.123800475	0.121730189	0.125850272	0.123496674	0.124356724	0.118883173	0.12606478	0.124466928	0.120672138	0.118113761	0.123829448
	2007	0.265462685	0.268419823	0.264510983	0.270166581	0.264177667	0.276187053	0.253719156	0.270625278	0.273312698	0.275972832	0.257166035	0.268477158
	2008	0.706580783	0.69850037	0.680787151	0.721079286	0.691086664	0.71207459	0.655344824	0.719060284	0.696559952	0.702999694	0.642699233	0.698784662
	2009	0.133267284	0.13404455	0.130789408	0.136176333	0.130869698	0.139857586	0.121813933	0.13709266	0.13838673	0.139973866	0.124803516	0.134091631
	2010	0.028217428	0.028706998	0.027179865	0.030167563	0.027667505	0.030610273	0.024485827	0.029974086	0.029504153	0.028552425	0.024391155	0.028728986
	2011	0.027041261	0.027315931	0.025459255	0.029109218	0.025784357	0.029995768	0.022689191	0.028741907	0.028206587	0.028659032	0.021709706	0.027344648
	2012	0.065026562	0.061177044	0.056274735	0.067981705	0.058739704	0.065441725	0.05158189	0.066340239	0.060428381	0.062269095	0.045127883	0.061259253
	2013	0.158825138	0.158061054	0.151358974	0.166000782	0.155634772	0.162306389	0.145046497	0.163361195	0.160689103	0.160816976	0.142952876	0.158138443
	2014	0.078185882	0.074679874	0.071557621	0.07965586	0.074337678	0.075278624	0.070982885	0.078169911	0.073035846	0.076303087	0.065978761	0.074724444
	2015	0.142657879	0.139028399	0.130557527	0.149705475	0.135731518	0.144797044	0.124396185	0.146328692	0.138584668	0.144589822	0.116500871	0.139143792
	2016	0.211721778	0.214954439	0.207687192	0.221785156	0.210298282	0.225234573	0.19558473	0.22057706	0.22253665	0.215546035	0.199073232	0.215036826
	2017	0.299019296	0.298745716	0.294970839	0.299596745	0.295530843	0.3058437	0.289413558	0.301902061	0.299143836	0.29803341	0.287481293	0.298804152
	2018	1.143420371	1.13855328	1.129419053	1.14504689	1.160711878	1.089630235	1.185399822	1.129209764	1.117740312	1.071686564	1.17911333	1.138342866

Exposure	Year	Population-Weighted Average Concentrations for Race and Ethnicity Groups (μg/m^3)											
		White Population	Non-White Population	Black Population	Non-Black Population	Asian Population	Non-Asian Population	Hispanic Population	Non-Hispanic Population	Native American Population	Non-Native American Population	Pacific Islander Population	Non-Pacific Islander Population
Total $PM_{2.5}$	2006	11.00127208	12.47864964	12.50347031	11.83721985	11.9596448	11.8638939	12.75807839	11.35175589	10.57377977	11.88152901	11.49915175	11.87747954
	2007	11.33416792	12.90911285	12.90661174	12.22711774	12.22521012	12.27286017	13.26202666	11.67508039	10.81576095	12.27281865	11.71851115	12.26877289
	2008	11.58429731	12.81085095	12.86207742	12.27642271	12.31653757	12.30974123	13.05685424	11.86694495	11.40034172	12.31439597	12.02233258	12.31165569
	2009	10.33376981	11.61264052	11.66762733	11.05531928	11.16004148	11.08097299	11.85191225	10.63871386	9.865680889	11.09615954	10.75870815	11.0922682
	2010	9.403041745	10.51431342	10.4846823	10.03482921	10.10420682	10.05478218	10.74379485	9.655200093	8.934726625	10.06577469	9.659543775	10.06254676
	2011	10.23061505	11.35831903	11.30983111	10.90420579	10.96853761	10.92026199	11.58508579	10.50664418	9.842419657	10.93076379	10.61842576	10.92790388
	2012	9.399363561	10.48457375	10.45041604	10.04683558	10.06463968	10.07001704	10.71797722	9.655302567	9.164982945	10.07261758	9.744616493	10.07047963
	2013	9.878688118	10.90671784	10.92226373	10.48922189	10.57676624	10.50342114	11.08814955	10.14646106	9.711585658	10.51626589	10.43929779	10.51358797
	2014	9.488969487	10.64101471	10.61470004	10.17573142	10.18902848	10.20189199	10.88950392	9.760217251	9.280869995	10.20353757	9.835634407	10.20149034
	2015	9.294569558	10.30391718	10.32391535	9.893729298	9.878373384	9.92379389	10.52152983	9.532281582	9.233927603	9.920180414	9.657580522	9.918617365
	2016	8.748409914	9.76894035	9.761135739	9.372903045	9.334237459	9.404039717	10.0087988	8.990957337	8.540876751	9.397138755	9.092684087	9.395199389
	2017	8.946991778	9.966969711	9.972182839	9.570394881	9.559874485	9.597727094	10.18991886	9.200412945	8.729664104	9.59540457	9.405840767	9.593016398
	2018	10.44546806	11.19239279	11.35554118	10.89277672	11.1054052	10.88698425	11.2373085	10.70866799	10.4008429	10.919891	11.2272819	10.91693775
NWF $PM_{2.5}$	2006	10.86331034	12.36460258	12.38947823	11.7128113	11.85193704	11.73773999	12.64423925	11.22203306	10.36618321	11.75807717	11.37328003	11.75368654
	2007	11.0671261	12.63974386	12.64030709	11.95856675	11.98066054	12.00094953	12.9829271	11.41300989	10.54872662	12.00439302	11.46362124	12.00030387
	2008	10.77100774	12.19141097	12.24077117	11.57313705	11.67383827	11.60308054	12.46420146	11.1055153	10.2826939	11.61763967	11.24056549	11.61345783
	2009	10.19775521	11.47995291	11.53473811	10.92120316	11.04257208	10.94450456	11.71405915	10.50693367	9.717379701	10.96217435	10.63600725	10.95818248
	2010	9.369619221	10.48885431	10.46032817	10.00585243	10.08125514	10.02523359	10.71810643	9.624698557	8.881873558	10.03716822	9.628510893	10.03384827
	2011	10.20101275	11.33242041	11.28638665	10.87666176	10.94728732	10.89200003	11.55752173	10.47948663	9.796176501	10.90351748	10.59224193	10.90058381
	2012	9.318026599	10.43589366	10.40366134	9.984808771	10.02362925	10.00569467	10.66896695	9.586360734	9.000383238	10.01182093	9.687806949	10.0092866
	2013	9.697511018	10.76298616	10.77769564	10.3303658	10.44610972	10.34108585	10.94279505	9.980290672	9.464247678	10.35853317	10.28485851	10.35551363
	2014	9.392914213	10.57958533	10.5506292	10.10042651	10.12657206	10.12530572	10.83187984	9.674652506	9.115713627	10.12919052	9.756092728	10.12682831
	2015	9.117248376	10.18862617	10.21292724	9.753048783	9.770060513	9.779974912	10.40883184	9.376449246	8.896459162	9.781881904	9.507854329	9.779628135
	2016	8.522436441	9.560382476	9.563886745	9.156925563	9.15110883	9.183807153	9.789872116	8.778608154	8.279892268	9.182347699	8.900358263	9.180163178
	2017	8.630899097	9.678293939	9.68897582	9.270751322	9.278486258	9.296102741	9.900478074	8.89556445	8.336094339	9.296995434	9.096841657	9.294307732
	2018	9.117233049	10.16395016	10.21164516	9.754532189	9.838268801	9.769755754	10.35249132	9.403698105	8.893512548	9.782646739	9.610088176	9.780114076
WF $PM_{2.5}$	2006	0.137961725	0.114046994	0.113992017	0.124408502	0.107707719	0.126153865	0.113839048	0.129722814	0.20759662	0.123451796	0.125871838	0.12379295
	2007	0.267041794	0.269368932	0.266304569	0.268550948	0.244549424	0.271910608	0.279099531	0.262070448	0.267034285	0.268425588	0.254889745	0.268468981
	2008	0.813289548	0.619439968	0.621306117	0.703285647	0.642699273	0.706660671	0.592652796	0.761429613	1.117647833	0.69675628	0.781766959	0.698197843
	2009	0.136014611	0.132687682	0.132889236	0.134116168	0.117469447	0.136468478	0.137853182	0.131780216	0.14830127	0.133985228	0.122701007	0.134085764
	2010	0.033422573	0.025459173	0.024354194	0.028976829	0.022951735	0.029548643	0.025688477	0.030501591	0.052853135	0.028606525	0.031032982	0.028698548
	2011	0.029602313	0.025898624	0.023444415	0.027544042	0.021250315	0.028261966	0.02756407	0.02715757	0.046243241	0.027246316	0.026183934	0.027320074
	2012	0.081336986	0.048680079	0.046754777	0.062026806	0.041010493	0.064322357	0.049010216	0.068941871	0.164599736	0.060796652	0.056809591	0.061193026
	2013	0.181177098	0.143731629	0.144568109	0.158856061	0.13065641	0.162335271	0.145354455	0.166170361	0.247338006	0.157732691	0.154439068	0.158074309
	2014	0.096055313	0.061429433	0.064070917	0.075304955	0.062456515	0.076586312	0.057624115	0.085564799	0.165156408	0.074347098	0.079541714	0.074662082
	2015	0.177321181	0.115291052	0.110988116	0.140680538	0.108312856	0.143819006	0.112698055	0.155832332	0.337468361	0.138298532	0.149726112	0.138989252
	2016	0.225973465	0.208557874	0.197249012	0.215977477	0.183128576	0.220232569	0.218926705	0.212349168	0.260984421	0.214791054	0.192325748	0.215036208
	2017	0.316092686	0.288675768	0.28320695	0.299643563	0.281388217	0.301624354	0.289440794	0.30484849	0.39356985	0.298409135	0.308999125	0.298708665
	2018	1.328235031	1.028442682	1.14389603	1.13824457	1.267136415	1.117228529	0.884817234	1.304969909	1.507330342	1.137244296	1.617193775	1.136823706

Exposure	Year	Absolute Difference in Population-Weighted Average Concentrations between Socioeconomic Groups ($\mu g/m^3$)					Absolute Difference in Population-Weighted Average Concentrations between Race and Ethnicity Groups ($\mu g/m^3$)					
		Unemployed and Employed	Below Poverty and Above Poverty	Without and With College Educational Attainment	Without and With High School Enrollment	Lower and Higher Income	White and Non-White Population	Black and Non-Black Population	Asian and Non-Asian Population	Hispanic and Non-Hispanic Population	Native American and Non-Native American Population	Pacific Islander and Non-Pacific Islander Population
	2006	0.042632016	0.780172774	0.649262163	0.31973454	0.583599385	-1.477377564	0.666250458	0.095750905	1.406322496	-1.307749237	-0.378327789
	2007	0.061777589	0.876118171	0.742061492	0.343938575	0.659807536	-1.574944937	0.679494002	-0.047650048	1.586946275	-1.4570577	-0.550261736
	2008	0.077743389	0.775791272	0.656848568	0.318340869	0.618049193	-1.226553639	0.585654711	0.006796344	1.189909297	-0.914054256	-0.289323111
	2009	0.031866652	0.680664057	0.532805418	0.269131592	0.509388663	-1.278870707	0.612308058	0.079068486	1.213198392	-1.230478647	-0.333560046
	2010	0.018922551	0.547838838	0.441600151	0.196114644	0.395732926	-1.111271676	0.449853091	0.049424644	1.08859476	-1.131048064	-0.403002985
	2011	0.012346048	0.57677596	0.479040403	0.202659275	0.461209389	-1.127703988	0.405625316	0.048275612	1.078441612	-1.088344131	-0.30947812
Total $PM_{2.5}$	2012	0.021285353	0.558062677	0.498876096	0.204636955	0.459268787	-1.08521019	0.403580462	-0.005377353	1.062674653	-0.907634633	-0.325863134
	2013	0.047755296	0.588603496	0.526369333	0.206785678	0.486922793	-1.028029724	0.433041837	0.073345098	0.941688492	-0.804680236	-0.074290182
	2014	0.026556237	0.616688448	0.561920272	0.20932183	0.511763934	-1.152045226	0.438968617	-0.012863513	1.129286666	-0.922667572	-0.365855936
	2015	0.059661819	0.595060632	0.547812592	0.20285963	0.504358643	-1.009347624	0.430186048	-0.045420506	0.989248253	-0.68625281	-0.261036843
	2016	0.02620509	0.520891247	0.488217501	0.071381969	0.457444603	-1.020530436	0.388232694	-0.069802258	1.017841467	-0.856262004	-0.302515302
	2017	0.026925931	0.549167692	0.509935233	0.081680354	0.469557955	-1.019977933	0.401787958	-0.037852609	0.989505915	-0.865740466	-0.187175631
	2018	0.01995669	0.436685409	0.378403163	0.031326262	0.395203354	-0.746924734	0.462764453	0.218420951	0.528640507	-0.519048102	0.310344158
	2006	0.038511927	0.779312682	0.642080514	0.323529312	0.57788367	-1.501292234	0.676666931	0.114197051	1.422206195	-1.391893956	-0.380406508
	2007	0.056121973	0.864108799	0.725155401	0.341278503	0.648496437	-1.572617758	0.681740341	-0.020288994	1.569917209	-1.455666394	-0.536682631
	2008	0.037451246	0.754803367	0.593133151	0.311901033	0.561963806	-1.420403231	0.667634116	0.070757727	1.358686163	-1.334945775	-0.372892337
	2009	0.026479728	0.671676188	0.517526749	0.267544634	0.500100579	-1.282197695	0.613534959	0.098067525	1.207125487	-1.244794653	-0.322175228
	2010	0.015934845	0.544896048	0.436111901	0.197066354	0.391395101	-1.119235088	0.454475739	0.056021554	1.093407878	-1.155294662	-0.405337375
	2011	0.008696061	0.572564489	0.472987684	0.202206867	0.455574456	-1.131407659	0.409724884	0.055287284	1.078035095	-1.107340982	-0.308341878
NWF $PM_{2.5}$	2012	0.009578371	0.551360608	0.484117685	0.202796327	0.443137365	-1.117867059	0.418852566	0.017934582	1.082606215	-1.011437689	-0.321479653
	2013	0.033113501	0.581931851	0.508054647	0.206657804	0.471737216	-1.065475142	0.447329847	0.105023868	0.962504376	-0.89428549	-0.070655123
	2014	0.018457991	0.615747474	0.55473323	0.206054608	0.503018215	-1.186671121	0.450202686	0.001266339	1.157227334	-1.013476889	-0.370735581
	2015	0.040513878	0.585995097	0.525880122	0.196854476	0.481715683	-1.07137779	0.459878453	-0.009914399	1.032382595	-0.885422742	-0.271773806
	2016	0.012107143	0.505954967	0.463225212	0.078372786	0.441480996	-1.037946035	0.406961181	-0.032698323	1.011263963	-0.902455431	-0.279804915
	2017	0.022300044	0.538854857	0.497446747	0.082790822	0.458235093	-1.047394843	0.418224498	-0.017616483	1.004913624	-0.960901096	-0.197466075
	2018	0.004328884	0.507767104	0.434593251	0.077380025	0.435973843	-1.046717108	0.457112969	0.068513047	0.948793217	-0.889134191	-0.1700259
	2006	0.004120082	0.00086005	0.007181607	-0.003794791	0.005715687	0.023914731	-0.010416485	-0.018446146	-0.015883766	0.084144824	0.002078888
	2007	0.005655598	0.012009386	0.016906122	0.002660134	0.011311123	-0.002327138	-0.002246379	-0.027361184	0.017029084	-0.001391303	-0.013579236
	2008	0.040292135	0.020987926	0.06371546	0.006439741	0.056085429	0.19384958	-0.081979529	-0.063961398	-0.168776817	0.420891553	0.083569116
	2009	0.005386925	0.008987888	0.015278727	0.001587136	0.009288115	0.003326929	-0.001226932	-0.018999031	0.006072967	0.014316043	-0.011384757
	2010	0.002987698	0.002942768	0.005488259	-0.000951728	0.004337832	0.007963401	-0.004622635	-0.006596908	-0.004813114	0.024246609	0.002334435
	2011	0.003649963	0.004211411	0.006052716	0.000452445	0.005634942	0.003703689	-0.004099627	-0.007011652	0.0004065	0.018996925	-0.00113614
WF $PM_{2.5}$	2012	0.011706971	0.00670202	0.014758349	0.001840714	0.01613137	0.032656907	-0.01527203	-0.023311864	-0.019931654	0.103803084	-0.004383435
	2013	0.014641808	0.006671617	0.018314698	0.000127873	0.015185568	0.037445469	-0.014287952	-0.031678861	-0.020815906	0.089605314	-0.003635241
	2014	0.008098239	0.000940946	0.007187026	0.003267241	0.008745683	0.03462588	-0.011234038	-0.014129798	-0.027940684	0.09080931	0.004879632
	2015	0.019147948	0.009065527	0.021932507	0.006005154	0.022642921	0.062030129	-0.029692422	-0.03550615	-0.043134276	0.199169829	0.01073686
	2016	0.014097964	0.01493629	0.02499233	-0.006990615	0.015963594	0.017415592	-0.018728466	-0.037103993	0.006577538	0.046193367	-0.02271046
	2017	0.004625905	0.010312857	0.012488504	-0.001110426	0.011322859	0.027416918	-0.016436613	-0.020236137	-0.015407696	0.095160715	0.01029046
	2018	0.015627837	-0.071081643	-0.056190058	-0.046053748	-0.040770464	0.29979235	0.00565146	0.149907886	-0.420152675	0.370086045	0.480370069
