# Supplementary material for: The diverging role of increasing wildfire smoke to ambient PM2.5 exposure disparity in California, 2006 to 2018
Source: PLOS Clim. Author manuscript; Available in PMC 2026 Jun 18. (PMC13274699; doi:10.1371/journal.pclm.0000796)
Supplement: S2_Table — S2 Table. Average sociodemographic indicators among census tracts least (≤10th percentile) and most exposed (≥90th percentile) to total, non-wildfire (NWF) and wildfire (WF) PM2.5 in averages of years 2006–2008 and averages of years 2016–2018. [file NIHMS2173162-supplement-S2_Table.pdf]

$PM_{2.5}$ Type	Indicators	Average sociodemographic indicators across census tracts (%)			
		2006-2008		2016-2018	
		Least exposed	Most exposed	Least exposed	Most exposed
Census tracts assorted via total $PM_{2.5}$	% employed	67.07	67.31	67.28	69.89
	% above poverty	66.10	50.73	66.25	56.67
	% with a Bachelor's degree	26.45	15.99	27.04	19.45
	% high school enrollment	97.41	96.13	96.61	97.41
	% non-Hispanic white	63.25	25.31	55.69	24.87
	% non-Hispanic Black	3.34	5.87	3.54	4.23
	% non-Hispanic Asian	3.57	10.15	3.43	12.04
	% Hispanic	26.00	56.44	33.15	56.30
	% non-Hispanic Native American	1.03	0.33	1.01	0.31
	% non-Hispanic Pacific Islander	0.23	0.19	0.22	0.25
Census tracts assorted via NWF $PM_{2.5}$	% employed	66.96	67.41	67.88	71.52
	% above poverty	66.42	50.49	67.70	59.22
	% with a Bachelor's degree	26.46	15.99	28.08	21.18
	% high school enrollment	97.35	96.03	96.82	96.91
	% non-Hispanic white	64.93	23.90	60.51	21.16
	% non-Hispanic Black	3.13	5.77	3.19	4.45
	% non-Hispanic Asian	3.46	10.89	3.50	14.01
	% Hispanic	24.53	57.29	28.20	57.96
	% non-Hispanic Native American	1.12	0.32	1.14	0.28
	% non-Hispanic Pacific Islander	0.22	0.17	0.22	0.26
Census tracts assorted via WF $PM_{2.5}$	% employed	67.91	66.23	73.32	71.07
	% above poverty	65.10	60.45	72.26	67.33
	% with a Bachelor's degree	26.22	21.45	36.50	28.67
	% high school enrollment	97.62	96.96	98.06	97.32
	% non-Hispanic white	48.40	57.50	44.38	52.39
	% non-Hispanic Black	5.28	3.33	4.49	6.13
	% non-Hispanic Asian	7.51	6.87	11.17	10.01
	% Hispanic	35.47	27.99	35.92	25.36
	% non-Hispanic Native American	0.49	1.11	0.32	0.76
	% non-Hispanic Pacific Islander	0.30	0.32	0.33	0.59
